# Measuring Encapsulation Efficiency in Cell-Mimicking Giant Unilamellar Vesicles

**DOI:** 10.1021/acssynbio.2c00684

**Published:** 2023-03-28

**Authors:** Pashiini Supramaniam, Zibo Wang, Stelios Chatzimichail, Christopher Parperis, Aditi Kumar, Vanessa Ho, Oscar Ces, Ali Salehi-Reyhani

**Affiliations:** † Department of Chemistry, Imperial College London, London W12 0BZ, U.K.; ‡ Department of Surgery & Cancer, Imperial College London, London W12 0HS, U.K.; § Department of Chemistry, King’s College London, London SE1 1DB, U.K.; ∥ fabriCELL, Imperial College London, London SW7 2AZ, U.K.; ⊥ Institute for Molecular Science and Engineering, Imperial College London, London SW7 2AZ, U.K.

## Abstract

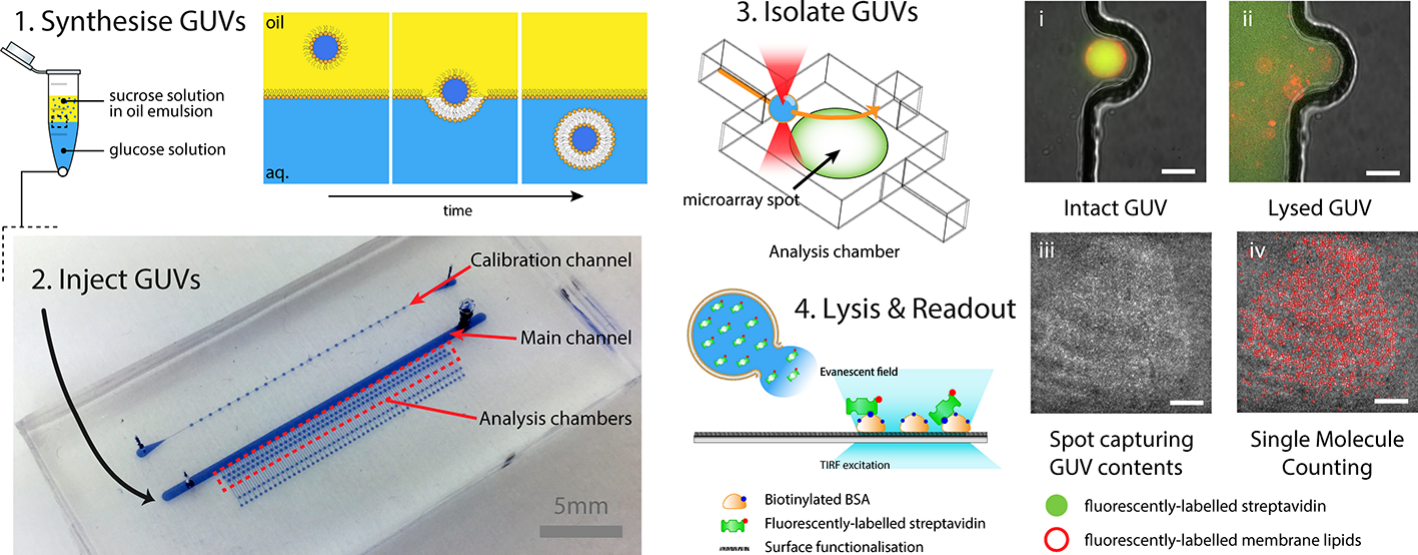

One of the main drivers within the field of bottom-up synthetic biology is to develop artificial chemical machines, perhaps even living systems, that have programmable functionality. Numerous toolkits exist to generate giant unilamellar vesicle-based artificial cells. However, methods able to quantitatively measure their molecular constituents upon formation is an underdeveloped area. We report an artificial cell quality control (AC/QC) protocol using a microfluidic-based single-molecule approach, enabling the absolute quantification of encapsulated biomolecules. While the measured average encapsulation efficiency was 11.4 ± 6.8%, the AC/QC method allowed us to determine encapsulation efficiencies *per* vesicle, which varied significantly from 2.4 to 41%. We show that it is possible to achieve a desired concentration of biomolecule within each vesicle by commensurate compensation of its concentration in the seed emulsion. However, the variability in encapsulation efficiency suggests caution is necessary when using such vesicles as simplified biological models or standards.

## Introduction

Bottom-up synthetic biology is capable of fabricating vesicles with cell-like features built from synthetic or biological constituents.^1−4^ Despite the wide range of options to synthesize cell mimics, quantitative methods to measure the contents of these artificial cells post-manufacture require development. This must be addressed for precision engineering of cell mimics to be an effective tool in the study of cell biology and as functional cell-like devices in the future. A key consideration in the production of cell mimics is the encapsulation efficiency (EE): to what extent molecules that are present in the seed (or loading) solution are encapsulated in the resultant vesicle? Loss mechanisms leading to lower encapsulation efficiencies have been suggested to include nonspecific adsorption of material at phase boundaries, vesicle rupture, and lack of access of seed solution to what becomes the vesicle interior before vesicles are formed.^1^ EEs can thus vary according to the method of vesicle production and the chemical nature of the molecule encapsulated.^5−7^ A wide range of factors that could contribute to a reduced encapsulation efficiency is a reflection of the sensitivity of the encapsulation toward the properties of the solute and vesicle alike. This therefore further highlights the importance of establishing a more quantitative method that allows the encapsulation efficiency to be determined experimentally. A low EE can pose problems for the application of cell mimics when encapsulating cellular biomolecules, often only available at low concentrations and in precious quantities. Critically, when a mixture of components is encapsulated (e.g., cell-free protein expression systems), different EEs of individual components may compromise the activity of the biosystem in question. Furthermore, vesicle-to-vesicle variations of EEs within a population of vesicles will in turn lead to a variation in performance.^8,9^ Precise measurement of EEs on a single-vesicle level is therefore desirable.

To date, there have been a handful of methods developed to measure encapsulation efficiencies, but these are either not suitable for the detection of proteins (only of fluorescent dyes) or are not performed at the single-vesicle level, instead relying on bulk averages. Colletier et al. investigated the encapsulation of the acetylcholinesterase enzyme into liposomes.^10^ They showed that the enzyme was rapidly denatured by most encapsulation protocols; lipid film hydration was able to preserve enzyme function but led to a very low encapsulation efficiency. Matosevic et al. demonstrated the stepwise synthesis of giant unilamellar vesicles (GUVs) using a microfluidic device incorporating flow-focusing and segregated fluid flow.^11^ By comparing the measured fluorescence intensity of the product vesicles post-phase transfer and the seed droplets pre-phase transfer, they could estimate the EE per vesicle, which they reported to be 83% and independent of droplet size. Göpfrich et al. on the other hand, introduced a one-pot method for the formation of complex single- and multicompartment synthetic cells relying on the formation of charge-mediated fusion of SUVs within surfactant-stabilized droplets.^12^ The use of this methodology reported high encapsulation efficiencies within the GUV. Sun et al. measured the EE of individual lipid vesicles encapsulating carboxyfluorescein prepared by rotary evaporation.^13^ Vesicles were ruptured by laser lysis, releasing encapsulated dye, which was then detected using fluorescence correlation spectroscopy performed within the vicinity of the ruptured vesicle. Data were then fitted to a model of diffusion to estimate the concentration within the vesicles prior to rupture and thereby estimate the EE. They showed that there was significant variation in encapsulation efficiency for multilamellar and oligo-lamellar vesicles with average EEs of 17.5 ± 8.9 and 36.3 ± 18.9%, respectively. Lohse et al. measured the EE of a small-molecule dye encapsulated into small unilamellar vesicles prepared using a standard rehydration procedure.^14^ They found that EE varied with an inverse relation as a function of vesicle size and plateaued at ∼15% for vesicles of 400–900 nm in diameter. Hussain et al. recently developed a range of methods, including reversed-phase HPLC and evaporative light-scattering detection, that enabled the direct quantification of encapsulated proteins within liposomes.^15^ They reported encapsulation efficiencies in the presence of neutral lipids ranging from 3 to 38%.

GUVs have been analyzed and processed using flow cytometry.^8,16,17^ Of particular note is the report by Matsushita-Ishiodori et al. who used imaging flow cytometry to characterize high-throughput populations of calcein-encapsulating vesicles produced by phase transfer. From the data, calcein intensity within similar-sized GUVs varied over several orders of magnitude, indicating the wide variation of EE of calcein in their methods and ∼1 to 3% of GUVs contained no detectable calcein.

An important set of discussions regarding the challenge of determining EE has been offered in a series of reports by Luisi, Stano, and collaborators.^18−21^ de Souza et al., in investigating the minimal size of cells capable of entrapping complete machinery of a cell-free expression system, found a significant enhancement, rather than loss, of encapsulation of macromolecular components.^18^ They modeled the entrapment of macromolecules as the cumulative probability of independent Poissonian events, but empirical observations deviated by several orders of magnitude from the statistical expectation. These suggested a possible super-concentration effect, which the authors hypothesized was due to an expulsion of water from the liposomes.

**Figure 1:**
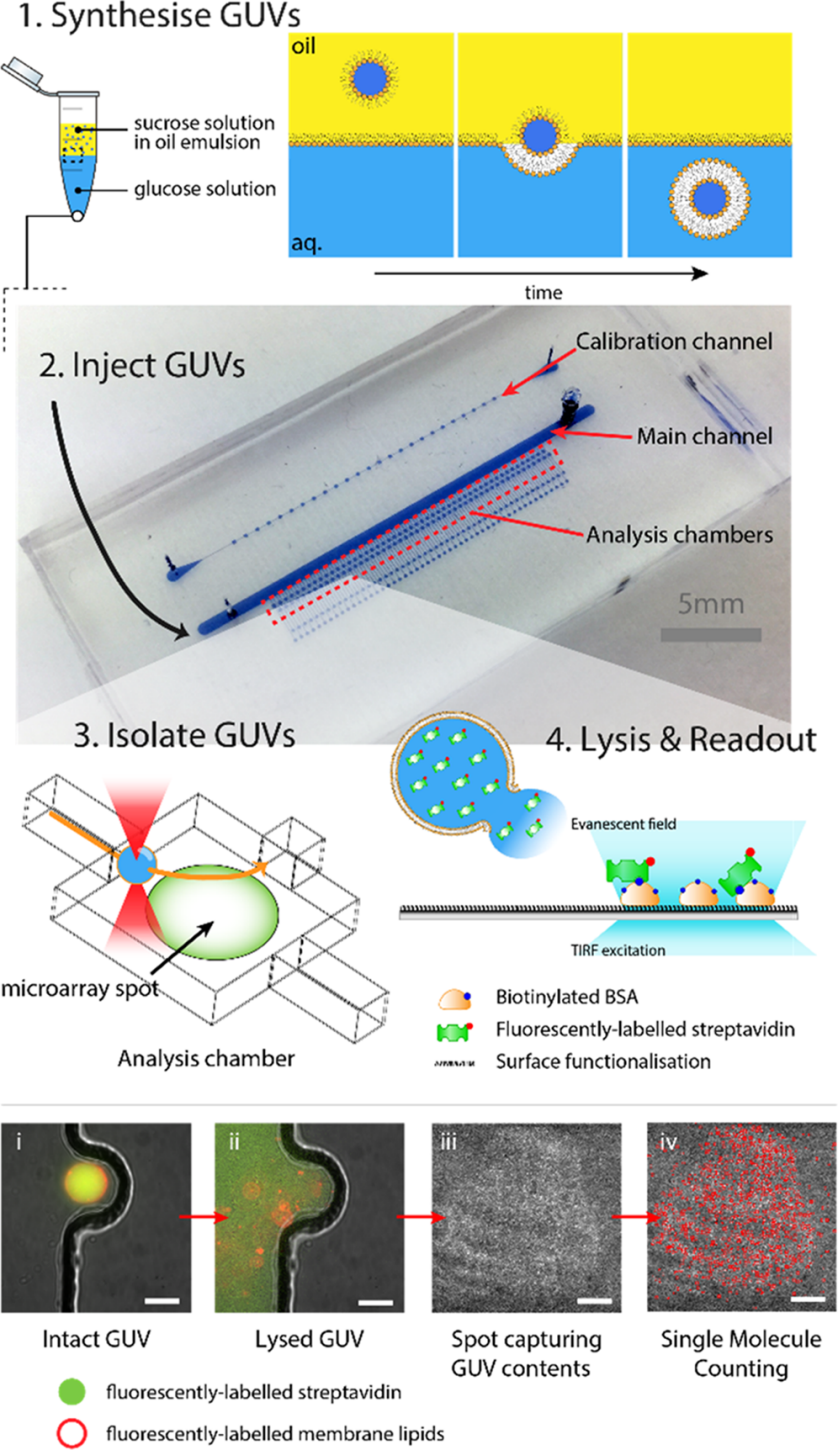
Schematic illustration of the single-molecule microarray microfluidic-based method to quantify biomolecules in GUVs. (1) GUVs encapsulating fluorescently labeled streptavidin are synthesized by phase transfer of an inverted emulsion and then (2) injected into a microfluidic chip; the device is composed of a main channel through which GUVs are flowed, connected by an array of reduced-volume analysis chambers. (3) An optical laser trap, capable of precisely manipulating GUVs, is used to isolate them into individual analysis chambers (image i). (4) GUVs are lysed by the action of a pulsed laser focused in their vicinity (image ii) and the released streptavidin AF488 is captured by a microarray spot of biotinylated BSA. Captured protein is imaged by single-molecule TIRF microscopy (image iii) and used to determine the number of streptavidin molecules per GUV (image iv). Image scale bar: 25 μm.

While these reports exist, they are easily overlooked in the growing body of literature and so the assumption that the encapsulated solution within a GUV has the same composition as that of the seed solution prevails. Here, we report a fluorescence-based single-molecule microarray approach to measure the absolute number of proteins within GUVs produced using phase transfer of an inverted emulsion (Figure 1).^22,23^ We use this to determine the EE of proteins within individual GUVs and preserve any variation that would be otherwise lost using bulk methods applied to heterogeneous GUV populations. The method presented is developed using labeled streptavidin and could be extended to other proteins and other biomolecules of interest.

## Results and Discussion

**Figure 2:**
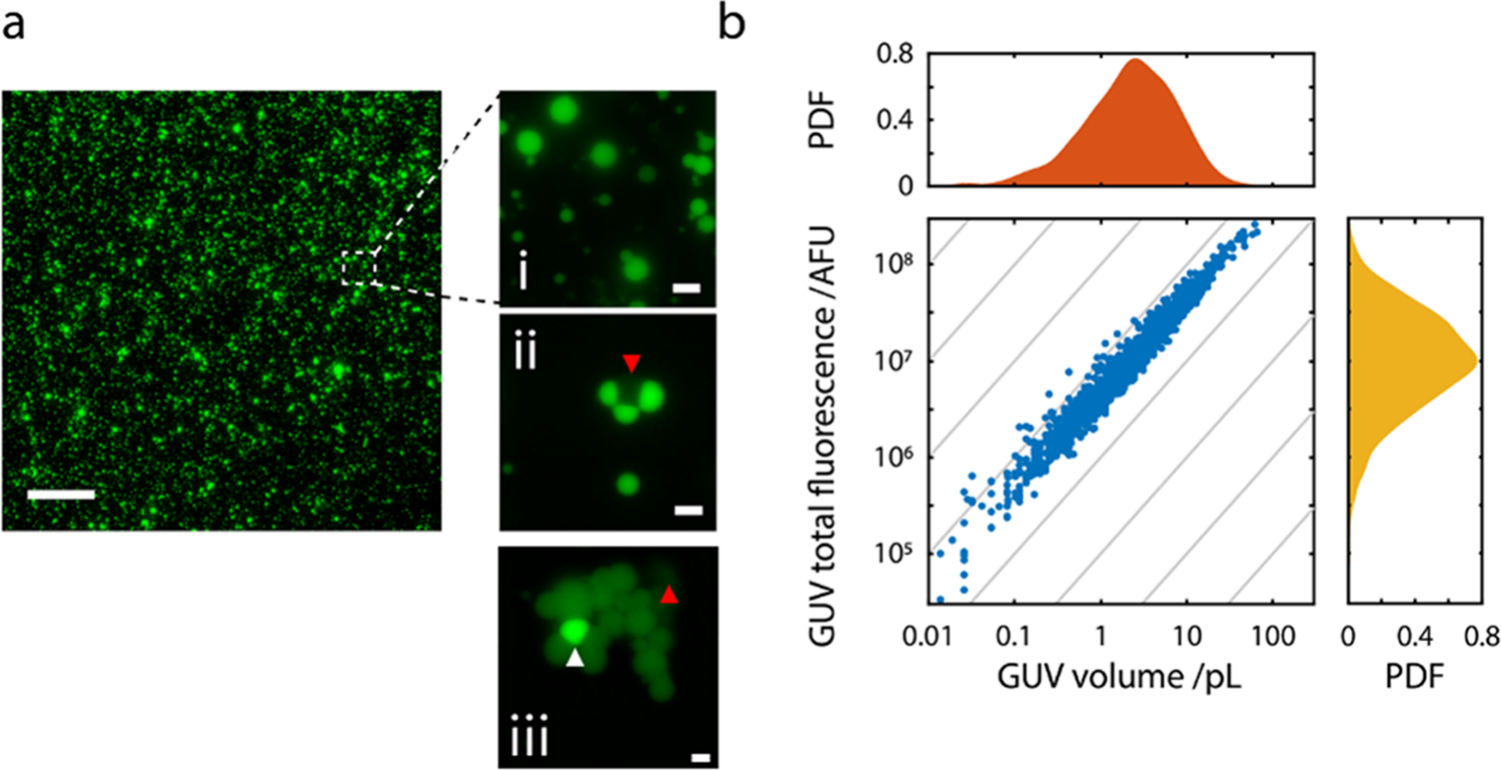
Assessing GUVs by fluorescence microscopy. (a) Large-field fluorescence microscopy of GUVs encapsulating fluorescent streptavidin AF488 produced by phase transfer. (i) Magnified area indicated by dashed white line to show the variation in GUV size and fluorescence intensity. (ii) Observation of a nonfluorescent *ghost* GUV (red arrowhead) semifused to fluorescent satellite GUVs. (iii) Observation of heterogeneous GUVs with both relatively low (red arrowhead) and high (white arrowhead) encapsulation efficiency. Scale bars (scale bars of magnified images): 250 μm (10 μm). (b) Image analysis identified individual GUVs (*n* = 2158) to measure their diameter, from which volume is calculated, and total fluorescence. Marginal histograms show the distributions of GUV volume (red) and GUV total fluorescence (yellow), respectively. The light gray guidelines follow lines of constant concentration, helping to indicate how encapsulation efficiency is constant with GUV volume. PDF, probability distribution function.

GUVs encapsulating streptavidin fluorescently labeled with Alexa Fluor 488 (AF488) are produced and imaged using fluorescence microscopy (Figure 2, see the Methods section for details). The encapsulation of streptavidin AF488 is confirmed by localized fluorescence within the extent of the lipid membrane. GUVs must be segmented in order to estimate their size and in turn measure their total fluorescence i.e., the sum of all segmented GUV pixels. From the raw images (Figure 2a) and histograms (Figure 2b) of the data, it is clear that the GUVs produced were heterogeneous in their size and total fluorescence. GUVs of varying encapsulation efficiency were observed; a good example of this is shown in Figure 2a, where similar-sized GUVs have significantly different fluorescence intensities. Some GUVs are also observed with little to no detectable fluorescent streptavidin AF488. An example is shown in Figure 2a(ii) whereby a “ghost” GUV only betrays its presence by the hemispherical shape of GUVs that are semifused to it. Time-resolved fluorescence microscopy indicates no evidence of leakage from the vesicles, so it is not clear how these ghost vesicles are themselves formed. It may be argued that it is improbable for a ghost vesicle to be the result of cargo leakage to the exterior medium, particularly in the case of large biomolecules such as proteins used here; pore formation to this degree would likely lead to total rupture. The fusion of GUVs, including protein-free membranes, can proceed under specific conditions.^24,25^ The coalescence of the membrane leads to the formation of semistable droplet interface bilayer (DIB)-like structures. In the absence of active pumping mechanisms in the membrane, the sharp asymmetry of fluorescence of neighboring GUVs here cannot be accounted for by the formation of pores through which proteins may pass. Nishimura et al. investigated the permeability of GUV membranes composed of POPC to small-molecule solutes, including amino acids and mononucleotides, oligonucleotides at the single-vesicle level.^26^ They found a small population of POPC GUVs with high permeability to charged and small molecules (<3 nm). From their results, there is an upper size limit on the size of permeable molecules since GUVs were impermeable to transfer RNAs (average molecular weight 27 kDa) and aminoacyl tRNA synthetases (average molecular weight 72 kDa). They considered the existence of a membrane defect area and estimated the size of a temporally stable pore to be ∼1.6 nm diameter. From these and other results, the POPC GUVs used here are unlikely to be permeable to the encapsulated labeled streptavidin Alexa Fluor 488 (average molecular weight 60 kDa), nor monomeric streptavidin. This would suggest that loss mechanisms are not associated with leakage via pores. da Silva et al. recently reported the generation of daughter vesicles from the bursting and reassembly of giant double emulsion droplets.^27^ They exploited this to incorporate a macromolecular fluorescent tracer (60 kDa bovine serum albumen conjugated to Alexa Fluor 647) present in the external medium. If this process proceeds in phase transfer using the conditions reported here, then incorporation of the external solution being buffer only would lead to a dilution effect consistent with our observations. da Silva et al. report the variation in encapsulation efficiency in the daughter vesicles with a modal EE of 60–70% and a small fraction (2%) of empty/“ghost” GUVs.

Noting the super-concentration effect reported by de Souza et al. in liposomes, we also observe GUVs with encapsulated fluorescent material significantly higher than that of neighboring GUVs (Figure 2a(iii)).^20^ It is unclear the processes by which these GUVs are produced here, and we note that they are infrequent in the populations produced here. It is feasible that such “super-concentrated” GUVs are the result of water expulsion through pores but would be expected to lead to non-spherical GUVs with low membrane tension. Nevertheless, these observations all indicate a significant variation of encapsulated material observed in these vesicles.

The total fluorescence intensity per GUV was plotted as a function of the GUV volume, as calculated from their measured diameters (Figure 2b). At face value, the gradient of the plotted logarithms of the data suggests that, to an extent defined by their variation, the concentration of streptavidin AF488 within the GUVs may be inferred to be broadly constant with vesicle volume. It is important to process the raw images appropriately, such as background subtraction. Of course, the fluorescence intensity is measured in arbitrary units and so such data can only provide relative measurements of the GUVs—it is not possible to calculate the absolute concentration of encapsulated streptavidin and by extension the encapsulation efficiency.

To determine the absolute number of streptavidin AF488 molecules encapsulated per GUV, we used a microfluidic-based single-molecule microarray approach. Microarrays are typically formed from thousands of small spots, on the order of 100 μm in diameter, where they react with specific analytes in a complex solution to perform a miniaturized biomolecular assay. By employing single-molecule microscopy as a readout for each spot, it is possible to achieve high sensitivity. Our approach involves four steps: (1) preparation of GUVs; (2) injecting these into a microfluidic chip; (3) isolating GUVs into individual analysis chambers using an optical trap; and (4) lysis of a GUV in the vicinity of a patterned microarray spot comprising of a suitable capture agent. The molecular cargo of a GUV which is released upon lysis binds to the capture agent and is detected using single-molecule fluorescence microscopy.

The microfluidic chip permits a reduced-volume microarray for the sensitive detection of biomolecules in single GUVs (see the Methodssection for details). The immobilized capture agent defines the class of molecule to be detected. Microarrays may be sensitive to DNA, RNA, and specific proteins using a variety of affinity-based capture agents. Here, we exploited the high binding affinity of streptavidin–biotin^28^ and used biotinylated bovine serum albumin (bBSA) as the capture agent (Figure 1, step 4). Spots of bBSA were microarrayed onto the surface of a coverslip that would serve to capture any encapsulated streptavidin released upon lysis of a GUV.

GUVs are introduced into the chip by a syringe pump. The microfluidic chip consisted of 100 analysis chambers each containing a single microarray spot. We have previously shown how optical tweezers can be used to sculpt biomimetic vesicle networks and manipulate liquid ordered lipid membrane domains.^29,30^ Here, an optical trap formed part of the microscope setup and was used to serially isolate individual GUVs into separate analysis chambers where they would be fluorescently imaged before being lysed by optically induced micro-cavitation.^31^ This method exploits a high-intensity laser pulse 10 μm above the GUV, which sets up an expanding bubble that mechanically shears it open. Although optical lysis necessitates a pulsed laser source, it offers precise optical control and avoids the use of detergents that may be incompatible with downstream assays. The encapsulated cargo of the lysed GUVs is now released into the analysis chamber for detection (Figure 1, see image ii). The encapsulated molecules diffuse into the analysis chamber and are captured by the microarrayed spot thereby depleting them from the chamber volume. The scaling advantages of nanoliter-scale chamber volumes reduce the time to achieve equilibrium and raise the effective concentration of analytes and their capture agents. The proximity of the capture spot to the lysed GUV, the diameter of the spot, and the aspect ratio of the chamber (∼1:10 height to lateral width) all strongly promote interaction with the spot. We have recently shown that the method used here can faithfully and precisely measure the distribution of fluorescence proteins from biological cells, and the equivalence between the single-cell microarray and immunofluorescence data demonstrates how the former may be used to achieve accurate absolute quantification.^32^

Microarray spots in each chamber were imaged using total internal reflection fluorescence (TIRF) microscopy (see the Methods section for details) and datasets were analyzed using a single-molecule detection algorithm to determine the number of streptavidin molecules originating from each GUV binding to each bBSA capture spot. The number of molecules counted per spot is dependent but not necessarily numerically equal to the number of molecules originally encapsulated by a GUV as a fraction will remain unbound. The microfluidic chip used to analyze GUVs additionally consists of a separately addressed calibration channel along which 25 chambers are placed with the same dimensions as the regular analysis chambers (Figure 1). It is possible to convert an on-spot single molecule count to an absolute streptavidin copy number by way of a standard calibration curve using solutions of streptavidin AF488 of known concentration (Figure S3, see the Methods section for details).

**Figure 3:**
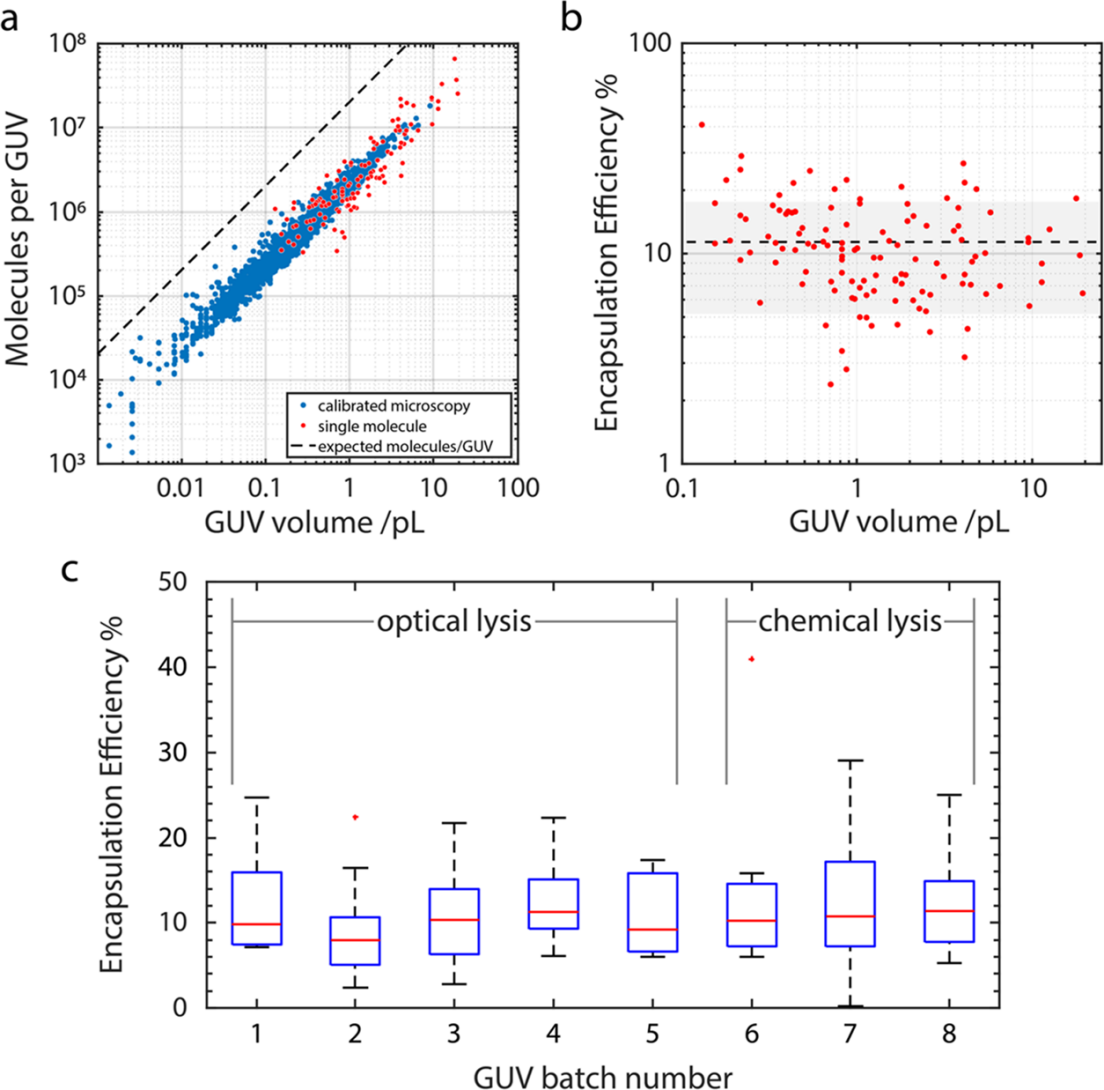
Absolute quantification to determine encapsulation efficiency. (a) Plot of the measured number of molecules per GUV and volume (red circles). The microscopy data (blue circles) is calibrated to absolute values using the single-molecule data. (b) Encapsulation efficiency is determined from the ratio of the measured to expected number of streptavidin proteins per GUV (*n* = 120 per batch). The dashed horizontal line indicates the mean EE (11.4%), and the gray box bounds the ± standard deviation (±6.2%) from the mean. (c) Box and whisker plots comparing the variation in EE for *n* = 8 independent batches, lysed using optical or chemical lysis methods. The horizontal red line is the median EE. Blue box extents bound the interquartile range (IQR), the lower quartile (25%) at the bottom, and the upper quartile (75%) at the top. The whiskers extend to the minimum value and the largest value not considered an outlier, i.e., upper quartile + 1.5 × IQR. The red circles indicate outliers.

The concentration of streptavidin AF488 in the originating solution from which the inverted emulsion is produced was 2.35 × 10^6^ molecules pL^–1^ (221 μg mL^–1^). The number of streptavidin AF488 molecules counted per GUV as a function of volume is shown in Figure 3a. There is clear consistency between the microscopy (blue) and single-molecule results (red). Now, since the absolute number of molecules encapsulated has been obtained, we are able to calculate an encapsulation efficiency per GUV.

The encapsulation efficiency was calculated from the ratio of the calibrated number of *measured* streptavidin molecules to the *expected* number of molecules, defined by the originating solution concentration and the volume of each GUV (Figure 3b). For all measured GUVs, the encapsulation efficiency spanned ∼1.25 orders of magnitude, varying from 2.38 to 41.0%, with an average of 11.4 ± 6.2%. This was tested over eight independently synthesized batches of GUVs, and the average encapsulation efficiency remained remarkably consistent at ∼10% (Figure 3c). While it appears in Figure 3b that there is a higher likelihood of measuring higher EE in GUVS with volume <0.5 pL, these are not statistically significant in this dataset and would require more pulldowns. However, it is worth noting the observations of Lohse et al. who reported higher EE for smaller liposomes.^14^ Though it is not clear whether EE is enhanced or the liposomes are a product of the super-concentration effects reported by de Souza et al.^20^

**Figure 4:**
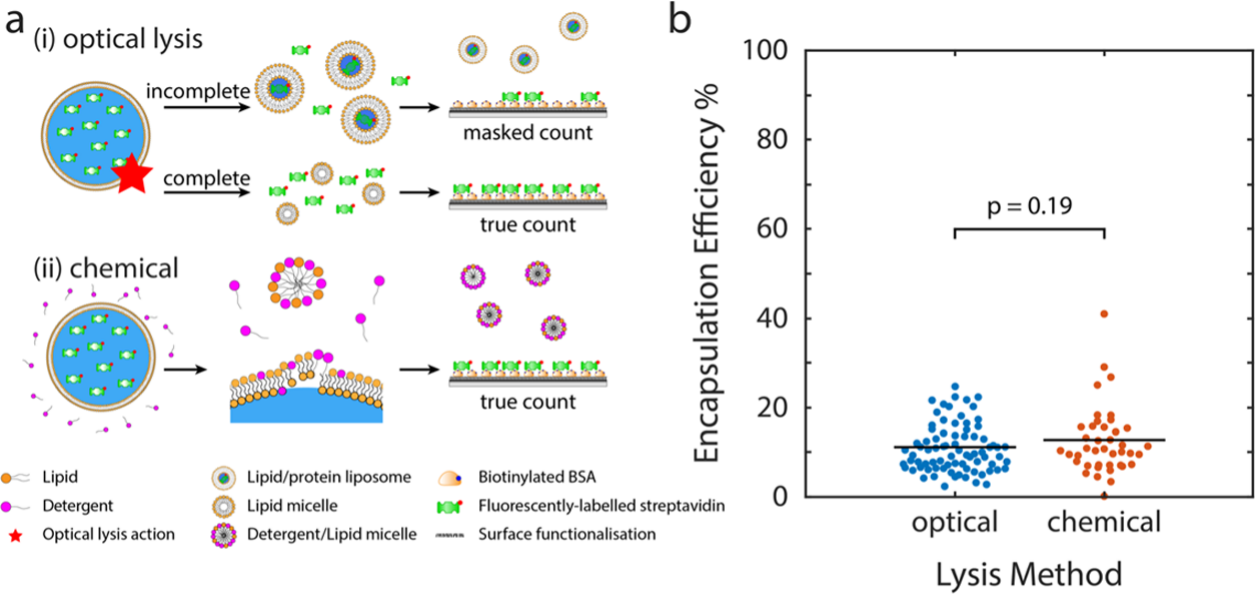
Comparison of optical and chemical methods to lyse GUVs. (a) Process of (i) optical lysis: The method uses a single pulse of laser that forms a cavitation bubble at close proximity to the vesicle, inducing shear stress which disrupts and compromises the membrane. This may result in the formation of smaller unilamellar vesicles which encapsulates GUV cargo shielding it from capture by the microarray; and (ii) chemical lysis: detergent destabilizes the membrane through incorporation into the lipid bilayer to form pores and eventually achieving full lysis. This allows for the full release of the protein within the vesicles. (b) No significant difference in the encapsulation efficiency is measured when lysing GUVs by optically induced micro-cavitation or flushing channels with RIPA buffer.

Optical lysis can under certain conditions induce fluorophore degradation. One example is if the wavelength of the lysis beam overlaps that of a fluorophore’s absorption spectrum. There is no measurable photobleaching caused by the delivery of a pulse from the optical lysis laser (1064 nm) for fluorophores used here (Alexa Fluor 488, peak absorption 490 nm).^32^ These results assume that the method of lysis is optimal and that GUV cargo is released fully into the aqueous solution of the analysis chamber and not retained within small unilamellar vesicles, therefore unavailable to bind to the sensing capture spot. Potentially, optical lysis may be ineffective in fully liberating the encapsulant materials into the bulk solution chamber. The optical method by which we lyse the GUVs appears to competently disintegrate them as observed by fluorescence microscopy (Figure 1, see image ii). Though, vesicles of sizes below the diffraction limit of the microscope may not be observed or be difficult to detect. To confirm this, we repeated EE experiments and instead chemically lysed the GUVs with a detergent (Figures 3c and 4). We chose radioimmunoprecipitation assay (RIPA) buffer since it is often used in cell biology for rapid, efficient cell lysis and solubilization of proteins from mammalian cells. These results suggest that the loss mechanisms are not a consequence of the method of lysis but of the method by which GUVs are produced (Figure 4).

**Figure 5:**
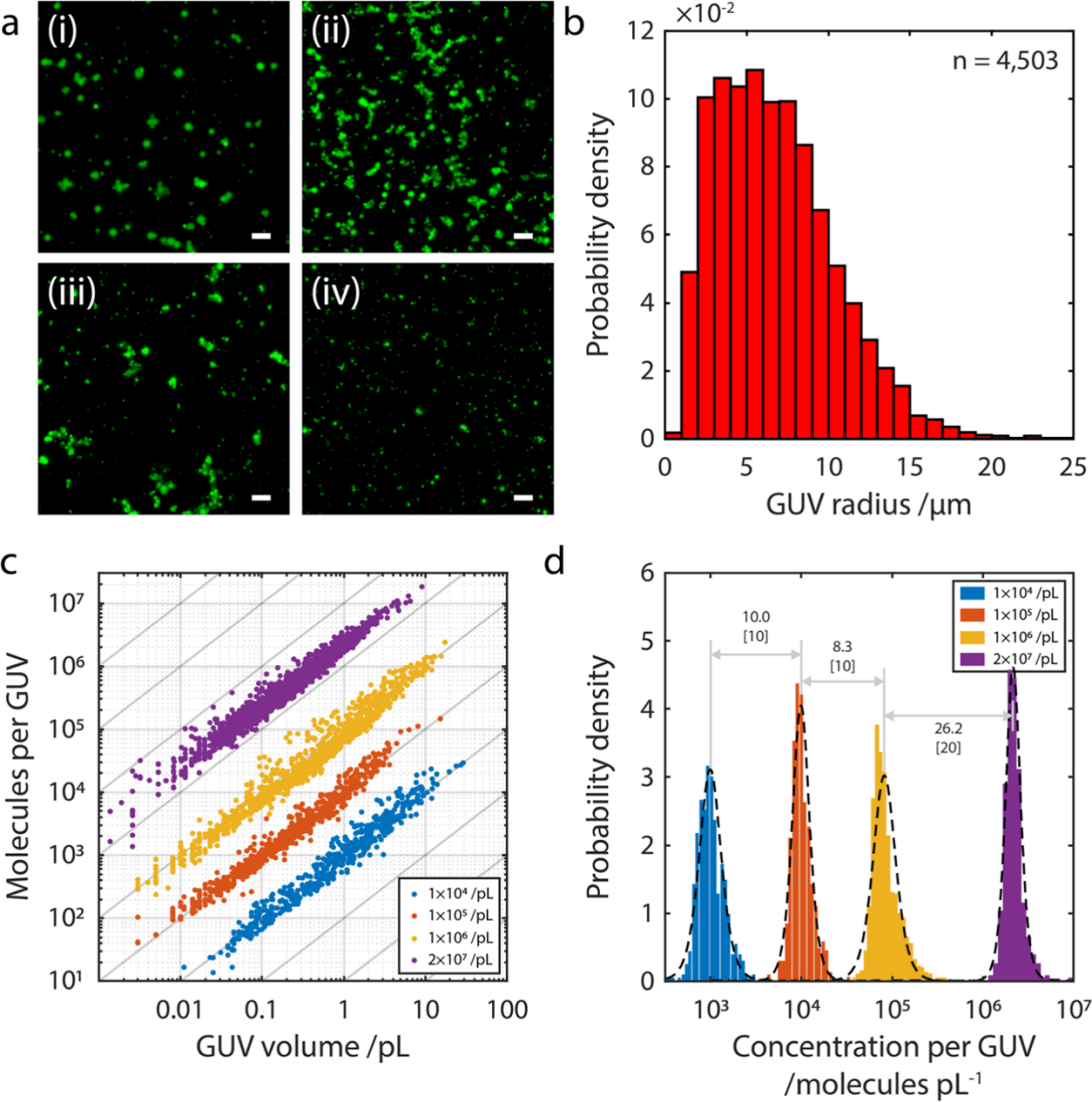
Producing GUVs with desired concentration of the protein cargo by compensating the concentration of the originating seed solution. (a) Fluorescence images of GUVs produced with seed solution concentrations of (i) 10^4^ proteins pL^–1^, (ii) 10^5^ proteins pL^–1^, (iii) 10^6^ proteins pL^–1^, and (iv) 2 × 10^7^ proteins pL^–1^. Scale bar: 100 μm. Note: contrast set to help visualize GUVs at each concentration. (b) Histogram of GUV radius for all batches. (c) Plot of the measured number of protein molecules encapsulated per GUV against GUV volume for GUVs prepared using different seed solution concentrations. (d) Histograms of the concentration within GUVs. Fits to each distribution are shown by dashed black lines. The numbers indicate the *measured* factor fold change in mean concentration compared with the *nominal* factor change in square brackets. (c, d) Concentrations in the legends are of the seed solutions used to produce GUVs.

One of the central tenets of bottom-up synthetic biology is the engineering-like control over a soft-matter system, in this case precisely controlling biomolecule copy number and behavior within an artificial cell. So, for our purposes of realizing simplified biological models, it is necessary then that if we have measured the EE of a protein within our GUVs to be significantly less than 100%, that we (i) optimize the method of phase transfer of an inverted emulsion or (ii) investigate whether we are able to compensate for low EEs to produce GUVs with a desired protein concentration. The relative consistency of the encapsulation efficiency across different batches would seem to suggest that altering the concentration of the originating seed emulsion may be effective. To test this, we re-prepared GUVs whereby streptavidin AF488 was present in seed solutions at 10^6^, 10^5^, and 10^4^ proteins pL^–1^ concentration. GUVs produced from each seed solution were assessed (Figure 5). The variation in the absolute number of protein molecules per GUV arises due to variations in GUV volume, which was largely consistent for all concentrations tested spanning ∼0.01 to 50 pL. As observed in Figure 5c, the absolute numbers of molecules per GUV may not be unique between populations of GUVs synthesized from different seed concentrations, e.g., GUVs of 0.01, 0.1, and 1 pL produced from 1 × 10^6^, 1 × 10^5^, and 1 × 10^4^ proteins pL^–1^, respectively, all contain, roughly, 10^3^ proteins. The orthogonal variation observed at each concentration and the factor difference between seed solutions are better assessed by calculating the concentration of protein molecules per GUV (Figure 5d). The distributions of protein concentration per GUV are separated with low overlap between the batches, indicating that 10-fold factor changes in concentration are readily achievable with phase transfer. 2-fold factor changes in concentration can be biologically important for the detection of copy number variation in disease, i.e., haploinsufficient gene expression. While factor 2 changes between populations of GUVs may be *distinguishable* in populations of GUVs with variance similar to the distributions in Figure 5d, there would be significant overlap of the distributions. They may be statistically distinguishable in separate batches, but difficult to separate if part of the same population of GUVs.

The results obtained here show that the use of GUVs as reported here as simplified cell models suggest that the presence of the variation in the encapsulation efficiency must be taken into account when analyzing and interpreting dynamic models. What could be deduced as heterogeneity in response could possibly be a result, or at least partly be a contribution of the molecular processes of encapsulation, or partial rupture after encapsulation or any other molecular mechanism that is not being accounted for and controlled in the production of the vesicles. These result in non-negligible variation of the EE per GUV that do not appear to be controlled by usual batch methods. However, it is arguably the orthogonal variation that exists within the set of populations that may pose a bigger problem. Microfluidic preparations have been reported to generate more homogeneous vesicles which may point toward the heterogeneity in solute distribution being a strong contributing factor to heterogeneity in the results reported here.^21^ Van de Cauter et al. showed that the EE and reproducibility of GUV formation were possible but through tightly controlling environmental conditions and tuning the dispersion of lipid in the oil phase.^33^ As in this report, dispersion of lipid in the oil phase is aided by creating lipid films using volatile solvents in the initial steps of preparation. Van de Cauter showed that the yield on nonfluorescent, or empty, GUVs was reduced from 23 to 10% when using decane-based lipid dispersion over chloroform-based lipid dispersion and necessary to work in a humidity-free glovebox to avoid products composed of residual membrane material. These data show that (for these experimental conditions) phase transfer is unlikely to achieve narrower distributions in protein encapsulant concentration and that any higher precision would likely need alternate methods of GUV production. Low encapsulation efficiency may be compensated for by altering starting concentrations of solutions; however, compensating for variation is not as straightforward, if at all possible, in some cases. As seen from Figure 5d, the variation in the concentration is similar in the GUV produced from both high concentration and low concentration. In lieu of methods demonstrating improved encapsulation efficiency, alternative strategies to achieving vesicles with more precisely defined compositions of biomolecules will be required.

**Figure 6:**
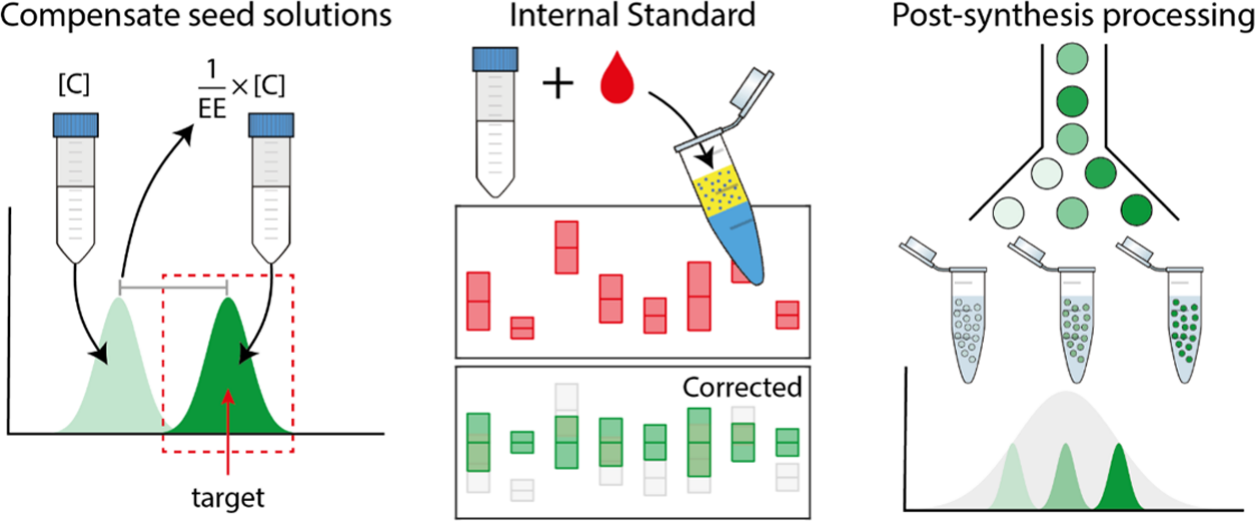
Proposed strategies to compensate for nonideal GUV synthesis. Compensation of seed solution to achieve the desirable encapsulant concentration. Use of spiked standard internal solution within the GUV to help correct for *intrinsic* variation in vesicle production by phase transfer. Processing GUVs post-synthesis may be carried out using fluorescence-activated cell sorting. The GUVs can be sorted based on a number of parameters, such as the concentration of the molecules within the vesicle, hence allowing for a tighter distribution and lesser overlap between the different populations.

While solutions to produce more homogeneous GUV populations, in terms of size parameters at least, exist, they are naturally more complex. Indeed, phase transfer has been seen to be an attractive method predominantly due to the minimal barrier to entry for researchers wishing to generate simplified models of gene expression using cell-mimicking GUVs. This is certainly recognized in some approaches developed to achieve multicompartment vesicles in a facile and accessible manner.^34^ A number of strategies exist to overcome the limitations of phase transfer (Figure 6). For example, the compensation of the seed solution concentration is able to achieve the desired effect in our case. This may not always be possible especially when working with expensive or even precious biomaterials, such as components of cell-free expression systems, e.g., ribosomes. In these cases, strategies that are common in analytical chemistry, i.e., inclusion of an internal standard may help to compensate for intrinsic variation when post-processing the data. Interestingly, Dominak and Keating showed that absolute EE and its variance may be improved when encapsulating polymers into GUVs formed by gentle hydration through the inclusion of specific cosolutes in the seed solution.^35,36^

Processing a sample of GUVs post-synthesis may also be of benefit. For example, employing fluorescence-activated cell sorting (FACS) may help to overcome limitations of synthesis by phase transfer to produce GUV populations with tighter distributions of protein concentration/expression.^37,38^ Cytometry facilities are common in biological departments and require minimal training to operate competently. One might expect the throughput and yield of a bulk phase transfer and FACS workflow to be considerable and help maintain the facile nature of the method and maintain as low a barrier to entry as possible. Size-based passive filtering has been reported using membrane filtration as well as microfluidic systems with high recovery rates.^39−42^ It may be possible to harness synthetic biology approaches to act as internal standards or to design a dynamic population of vesicles that post-synthetically develop programmatically toward homogeneous expression. A report by Nourian and Danelon provides an intriguing example of how this might be achieved using models of gene expression but also caution when implementing such approaches in vesicle-based systems since only a small fraction of bulk DNA is encapsulated in a transcribable manner.^43^

## Conclusions

In summary, we have developed the AC/QC method, a single-molecule microarray-based approach to measure the molecular content of vesicle-based artificial cells or cell mimics. Using AC/QC, we measured the encapsulation efficiency of GUVs containing streptavidin protein produced by bulk phase transfer of an inverted emulsion, a facile technique to produce GUVs with high yield. We have demonstrated the technique using a model protein system; however, we recognize the limitations of the proposed method. For instance, the technological barriers which challenge widespread adoption, i.e., microfluidic chip fabrication, optical trapping, and TIRF microscopy. The focus of this manuscript was to develop quantitative methods which, currently, requires the use of specialized methods. While microarrays are predominantly used for capturing DNA or protein, they are adaptable to label-free proteins using an antibody sandwich assay or any class of biomolecule for which exists a suitable capture agent, for instance, small-molecule drugs or click-based chemistries. Over the last decade, antibody microarrays intended for single-cell analysis have helped with the availability of commercially available antibodies with high affinity. Proteins that are typically used in constructing artificial cells, particularly cytoskeletal proteins, are not a problem in this regard. Other membrane-bound protein complexes, such as membrane pore complexes, will be more challenging. Weaker affinity agents would impact several analytical parameters, most notably the limit of detection, i.e., the concentration of molecules within a GUV. This again highlights the need for techniques and methods to quantitatively QC artificial cells and their constituents.

However, the low amount of material encapsulated by a cell-sized GUV poses major analytical challenges for all molecule classes, and while label-free sandwich assays can broaden applications, no nonfluorescent biomolecules inevitably add to the burden of developing analytically sufficient antibodies and their optimization for use in microarray-based assays.

As noted by a commentary by Stano, the development of phase transfer for the preparation of GUVs has been revolutionary in the field of bottom-up artificial cells.^44^ Yet, fundamental questions concerning the mechanistic details of their formation still remain open. Over the last decade of artificial cell research, reports have overwhelmingly focused on the qualitative construction of artificial cells. For many observational studies that use few (e.g., *n* = 3) GUVs, there are sufficient GUVs within a batch, or population, with sufficiently similar characteristics to avoid significant variation or outliers altogether. It is clear, however, that even for the simple measure of protein concentration or copy number within a GUV, there is variation that is not being sufficiently accounted for. Measuring this variation is important, but understanding it will be key to developing more precise control over these entities and is likely to become critical in more biological and therapeutics-facing applications, e.g., vaccines, targeted drug delivery, and biologics. While we are focused on uniformity and control for biotechnological applications, the heterogeneity of conditions within the process of phase transfer will likely help toward a broader mechanistic understanding of the compartmentalization of macromolecules within vesicles. The results here do not explain these mechanisms, but we expect will help contribute to the continuing development of methods to quantitatively determine EE as well as the wider conversation on compartmentalization.

Producing artificial cells and controlling them at the molecular level is an immense and unprecedented challenge with the potential to transform future healthcare and therapeutics. The methods reported here can serve to precisely quantify the biomolecular content of these systems with single-molecule resolution and will be a useful tool in their optimization and design. Understanding the mechanisms which lead to nonideal encapsulation efficiencies, whether these are losses or enhancements, will be crucial to optimizing vesicle production in producing well-defined cell mimics. Nevertheless, this and other reports highlight that the principles by which artificial cells are engineered are not fully elucidated or understood, even for seemingly “simple” systems.

## Methods

### Synthesizing Giant Unilamellar Vesicles (GUVs)

The GUVs were formed using phase transfer of an inverted emulsion, where aqueous droplets are stabilized by lipid monolayers. Briefly, we emulsify a solution of streptavidin conjugated with Alexa Fluor 488 (AF488) in 200 mM sucrose in oil containing POPC lipid. As the droplets traverse the oil-water phase boundary where a second POPC lipid monolayer is assembled, a bilayer encases the droplet and defines the vesicle. The oil phase consisted of 1.0 mg mL^–1^ 1-palmitoyl-2-oleoyl-*sn*-glycero-3-phosphocholine (POPC) lipid in mineral oil. The internal aqueous, or seed, solution contained streptavidin protein fluorescently labeled with Alexa Fluor 488 in 200 mM of sucrose in phosphate-buffered saline (PBS) solution, while the external aqueous solution was made up of 200 mM glucose in PBS. The concentration of streptavidin AF488 in the seed solution is 2.04 × 10^7^ molecules pL^–1^. To dissolve the lipid in oil, the lipid was first dissolved in chloroform before removing the organic solvent using a stream of nitrogen. The film was left in a lyophilizer for 1 h to ensure all chloroform had been completely evaporated off. 1.0 mL of mineral oil was added to the lipid film prior to ultrasonication for another 30 min. 250 μL of the oil/lipid mixture was mixed with 25 μL of the seed solution and vortexed in short bursts. The water/oil emulsion was pipetted into a micro-centrifuge tube containing 150 μL of 200 mM glucose in PBS solution. The mixture was centrifuged for 30 min at 9000*g*. The oil layer was gently removed, and the aqueous phase was resuspended in 150 μL of external aqueous solution. The resulting mixture was centrifuged at 6000*g* for 7 min, and the supernatant was removed. This step was repeated three times to help reduce background fluorescence from nonencapsulated material. The addition of sucrose and glucose to the internal and external solutions, respectively, increased the relative density of the GUVs allowing them to readily sediment for imaging. Bovine serum albumin (BSA) present in solution is used as a blocking agent in many single-molecule experiments, capable of limiting nonspecific binding of proteins and helping to reduce limits of detection. Typically, 4% BSA in PBS (PBSA) buffer fills the microchannels of the chip to block all surfaces. GUVs were found to prematurely rupture in buffer containing BSA (Figure S1). To prevent any rupture and maintain a minimal background of nonencapsulated streptavidin, BSA was not added to the external aqueous solution. To limit nonspecific binding, chips were pre-blocked with 4% PBSA.

### Surface Modification and Preparation of Coverslips

Coverslips were either commercially sourced pre-modified (Nexterion coverslips; Schott, U.K.) or prepared in-house. We have previously shown the desirable properties of PEG polymers, well known for their properties in limiting nonspecific binding, in functionalizing coverslips to support single-molecule microarrays with high dynamic range.^45,46^ However, and despite the net neutral charge of the POPC lipid at pH 7.4, when introducing GUVs into the main microfluidic channel, they tended to adhere to the PEG-coated Nexterion coverslip surface. This prevented the GUVs from being isolated into individual analysis chambers using an optical trap and could not be overcome with an increase in optical trapping power. In response to this, we found that BSA-coated coverslips were able to prevent GUV adhesion. These were tested for their ability in supporting a single-molecule microarray of biotinylated BSA spots by establishing calibration curves for each of the different surface modifications (Figure S3). The capture spots on the BSA-coated coverslips reach binding saturation at ∼10^7^ molecules, roughly an order of magnitude lower than for the PEG-modified coverslips. However, the degree of nonspecific binding improved when using BSA-coated coverslips, nearly 3 times lower than in the case of the PEG-based coverslips resulting in a lower limit of detection. As such, the limit of detection using BSA-coated coverslips was 26 ± 10 streptavidin molecules per analysis chamber. Additionally, the GUV suspension buffer contains 200 mM glucose. The presence of glucose in the buffer had no observable effect on the binding activity between streptavidin AF488 and biotinylated BSA. To prepare BSA-coated coverslips, glass coverslips were placed in a container and rinsed with ultrapure water (18.2 MΩ cm) three times, followed by 96% ethanol and ultrapure water once again. The coverslips were then ultrasonicated with 1 M KOH for 20 min. Upon completion, the coverslips were washed with distilled water followed by 96% ethanol. The container was filled with 96% ethanol and sonicated for a further 20 min. The coverslips are then dried using a stream of nitrogen. 1.0 mL of 4% PBSA is pipetted onto the coverslip surface and then placed in an oven at 60 °C for 120 min. The coverslips were washed with ultrapure water, dried using nitrogen, and then stored at 4 °C before being used.

### Microarray Printing

Due to the high binding affinity of streptavidin to biotin, biotinylated BSA (bBSA) protein was used as the capture agent in the microarray spots. The spots were printed using a contact microarrayer (Omnigrid Micro; DigiLab, U.K.) and a 946MP2 stealth pin (ArrayIt). The microarray print buffer consisted of 3× saline sodium citrate buffer, 1.5 M betaine supplemented with 0.01% SDS. The printing solution consisted of a 1:1 mix of print buffer and biotinylated BSA solution; the concentration of the biotinylated BSA in the printing solution was 0.5 mg mL^–1^. The microarray pin was cleaned by ultrasonication for 20 min in a surfactant-based cleaning solution (ArrayIt), rinsed with ultrapure water, and then dried with a nitrogen gun. Spots are printed in locations defined by the locations of the microfluidic analysis chambers. The printed coverslips are stored at 4 °C before use.

### Microfluidic Chip Fabrication

Chips used for experiments were fabricated using well-established methods of soft lithography of PDMS (Sylgard 184, Dow Corning).^47^ Pre-polymer and a polymerizing agent were mixed using a ratio of 10:1, poured over the mask, and then degassed for 10 min to remove air bubbles. PDMS was left to cure for over 48 h on a flat surface before being cut and peeled off the mask. Each chip (Figure S2) consisted of a main channel (1 mm × 35 mm × 32 μm; width × length × height) connected via side channels to 100 individual analysis chambers (300 μm × 300 μm × 32 μm) resulting in individual assay volumes of 2.9 nL. In addition, a calibration lane with 25 analysis chambers was used to assess the performance of the microarray using standard solutions of streptavidin AF488 of known concentration. The chips were washed with ethanol and air-dried with nitrogen. The microchannels were sealed with a cover glass on which microarray spots were printed. The printed biotinylated BSA spots were aligned to the chambers using a home-built translation stage. When carrying out single-cell experiments, the cover glass is not plasma-bonded as it may strip away the functionalized layer on the surface. The adhesion between the PDMS chip and glass is sufficient to handle the flow rates used in the experiments without delaminating. Two pieces of square PDMS pieces were cut and plasma-bonded to each of the inlets. These were used as solution reservoirs.

### Experimental Platform

An inverted microscope (Nikon Ti-E or TE2, Nikon, Japan) was used as the experimental platform. The positions of the spot within each chamber were logged using an encoded XY stage (Nikon, Japan). The reservoirs that were bonded onto the inlets were filled with 4% PBSA in 200 mM glucose. The chip was degassed for 5 min in a desiccator chamber. The chip is mounted onto the microscope stage and secured into position and connected with tubing attached to a microfluidic pump (Labsmith). The syringe is used to draw solutions containing GUVs or protein standards through the channels of the microfluidic device that are pipetted into the inlet reservoir. Typically, PDMS is irreversibly bonded to glass by exposing both surfaces to a plasma prior to contact. This is not possible for the devices used here owing to the microarray spots. To avoid delamination of the device, the flow rate was kept at 2.5 μL min^–1^. To fill the main channel with vesicles, 10 μL of the GUV solution was drawn through the channel. GUVs were manipulated by optical trapping, formed using a continuous wave Ytterbium fiber laser (YLM-5, IPG Photonics, U.K.). GUVs were ruptured by optical lysis achieved by delivering a single pulse from a Nd:YAG laser (Surelite SL I-10, Continuum) in the vicinity of a vesicle. Upon lysis, the contents of the GUVs were released into the analysis chamber allowing the streptavidin molecules to bind to the biotinylated BSA spots. All spots in the microarray were imaged in sequence by total internal reflection fluorescence (TIRF) microscopy using an EM-CCD camera (IXON DU-897E, Andor Technologies, Ireland) to achieve single-molecule resolution. A solid-state 488 nm CW laser (Vortran) served as the TIRF excitation source. Images were acquired every 30 min for 2 h, ensuring that binding equilibrium on the spots had been achieved. A single molecule counting algorithm was used to analyze the TIRF images obtained pre- and post-lysis

### Image Analysis (Single-Molecule Counting)

Single-molecule images (512 × 512 pixels) obtained by TIRF microscopy were analyzed using algorithms written for ImageJ/Fiji.^48^ Raw images are field-flattened and background-subtracted to produce images from which single molecules were detected by fitting intensity peaks to an isotropic 2D Gaussian function. The Gaussian parameters of the single molecules in an image were estimated and optimized by least squares fitting. Peaks within a threshold for their size and intensity were considered as single molecules and contribute to the single molecule count per image frame. For microarray spots where the density of single molecules is relatively high, single molecules were no longer individually distinguishable. In this regime, the number of single molecules was instead estimated by dividing the total spot intensity by the average single-molecule intensity.

### Calibration of Single-Molecule Microarray Data

A standard curve for calibration was obtained to determine the performance of the biotinylated BSA microarray to capture recombinant streptavidin protein labeled with Alexa Fluor 488. A series of standard solutions were flowed into the calibration channel of the microfluidic chip. Concentrations were chosen such that a known number of streptavidin molecules occupied each analysis chamber, ranging from 10 to 10^9^ molecules per chamber (Figure S3). Upon flowing each standard solution through the calibration channel, spots were allowed to reach binding equilibrium. The low volume (*V*_chamber_ = 2.93 nL) and the high binding affinity of streptavidin to biotin resulted in a high fraction of the total target being bound. The level of nonspecific binding when using BSA-coated coverslips was 26 ± 10 molecules per image frame, or (1.4 ± 0.5) × 10^–3^ molecules μm^–2^. Using the calibration curve, the number of single molecules counted on the spot, *N*_SM,_ can be *converted* into number of proteins per chamber and by extension the number of proteins encapsulated in a GUV, *N*_prot_. A five-parameter model was fitted to the calibration data and used to calculate the number of proteins encapsulated per GUV from each single molecule count. The form of the five-parameter model is shown in eq 1, where *N*_SM_^bkd^ is the number of single molecules counted at background, *N*_SM_^sat^ is the number of single molecules counted at the saturation limit of the antibody spot, *m* is the slope parameter of the response, *c* is an inflection point parameter, and *A* is a parameter which controls asymmetry of the response toward the background and saturation asymptotes. Equation 1 is used to obtain data from the calibration curve.
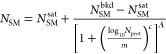
1

There are several critical components to the performance of this method. The EE is derived from the volume of the GUV and the number of molecules, calculated using a calibration curve from a single molecule count of antigens captured on a microarrayed spot. The printing of protein microarrays of high quality and reproducibility is crucial. How the analytical performance of miniaturized microarrays may be optimized in terms of assay and vessel design as well as optimal surface modification and printing buffers have been covered elsewhere.^49−52^ The quality of the microarray as well as the accuracy of enumerating bound antigens using single-molecule image analysis are important in establishing the calibration curve. The design of the microfluidic and microspots influences the dynamic range of the assay. It is possible to calibrate single molecule counts in the nonlinear regions of the curve but should be avoided to minimize uncertainty.

### Image Analysis (Wide-Field Fluorescence Microscopy)

Large-field fluorescence images were obtained by performing tile scans of 20 × 20 fields of view (∼2.66 mm × 2.66 mm) using a software-controlled motorized stage and imaged using a 60× NA = 1.49 oil immersion objective. Acquiring large image fields allowed a large sample of GUVs to be measured with individual GUVs identified by manual and automated image analysis methods. Images were background-subtracted, and image analysis was performed using FiJi (manual segmentation) and CellProfiler v2.2.0 (automated segmentation).^53,54^ The software is typically employed for identifying and quantifying cell phenotypes. Here, we apply similar methods to the analysis of GUVs. For each identified GUV, the radius and the total fluorescence intensity were determined by summing all pixel intensities for each identified GUV. Accurately determining the boundary of a GUV between the vesicle interior and exterior can be challenging in the fluorescence channel due to the fluorescence halo. We acquired image fields in both fluorescence and bright field. Bright-field images of GUVs were used to determine the accuracy of the segmentation performed in the fluorescence channel (Table S1). Since the estimation of GUV size was a critical parameter to calculating EE, data were processed manually given the high-degree heterogeneity of the GUVs (wide variation in size and intensity). The total fluorescence per GUV is calculated by the sum of the fluorescence of all segmented pixels of each GUV. Data plotting and further analysis were performed using MATLAB. To calibrate the microscopy data (Figure 2b) from arbitrary to absolute units, it was fit by the function *I*_μ_ = *a*_μ_*V*^*k*^, where *I*_μ_ is the measured fluorescence intensity per GUV, *V* is the GUV volume, and *a*_μ_ and *k* are constants. Similarly, the single-molecule data were fitted to *N*_SM_ = *a*_SM_*V*_k_, where *N*_SM_ is the number of streptavidin proteins measured per GUV, and *a*_*SM*_ and *k* are constants, where *k* is numerically equal in both cases. The quotient of these functions provides the ratio *a*_SM_/*a*_μ_, which was used to scale the microscopy data to absolute units of number of molecules (Figure 3a).

## Data Availability

All relevant data are available from the corresponding author upon reasonable request.

## Supporting Information Available

Results of suspending GUVs in solutions with differing BSA concentrations; schematic of the microfluidic device; calibration curve to calibrate single-molecule counts; and estimation of GUV volume based on different methods of image segmentation (PDF)
