# Supplementary material for: Measuring Encapsulation Efficiency in Cell-Mimicking Giant Unilamellar Vesicles
Source: ACS Synth Biol. 2023 Mar 28;12(4):1227–38. doi: 10.1021/acssynbio.2c00684 (PMC10127275; doi:10.1021/acssynbio.2c00684)
Supplement: Supplementary file 1 — sb2c00684_si_001.pdf [file sb2c00684_si_001.pdf]

# Supplementary Information:

# Measuring encapsulation efficiency in cell-mimicking giant unilamellar vesicles

Pashiini Supramaniam,[1] Zibo Wang,[2,3] Stelios Chatzimichail,[2] Christopher Parperis,[1,3] Aditi Kumar,[1] Vanessa Ho,[1] Oscar Ces,[1,4] and Ali Salehi-Reyhani[2,4,5]*

[1] Department of Chemistry, Imperial College London, London, W12 0BZ, UK

[2] Department of Surgery & Cancer, Imperial College London, London, W12 0HS, UK

[3] Department of Chemistry, King's College London, London, SE1 1DB, UK

[4] fabriCELL, Imperial College London, SW7 2AZ, UK

[5] Institute for Molecular Science and Engineering, Imperial College London, SW7 2AZ, UK

* corresponding author: ali.salehi-reyhani@imperial.ac.uk

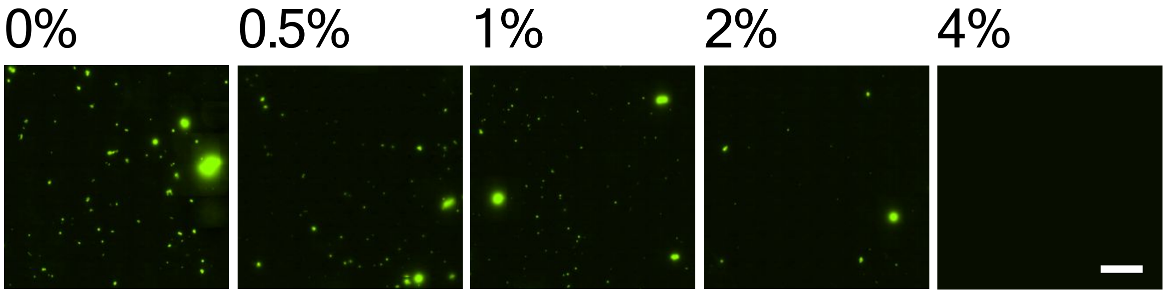


**Fig. S1:** GUVs encapsulating streptavidin Alexa Fluor 488 (green) were resuspended in solutions containing the denoted percentage (w/v) of BSA in 200 mM glucose in PBS. Images depict the degree to which GUVs rupture in the corresponding solutions. Minimal rupture was observed with 0% BSA, therefore upon production GUVs are resuspended in solutions of 200 mM glucose in PBS only. Scale bar: 500 μm.

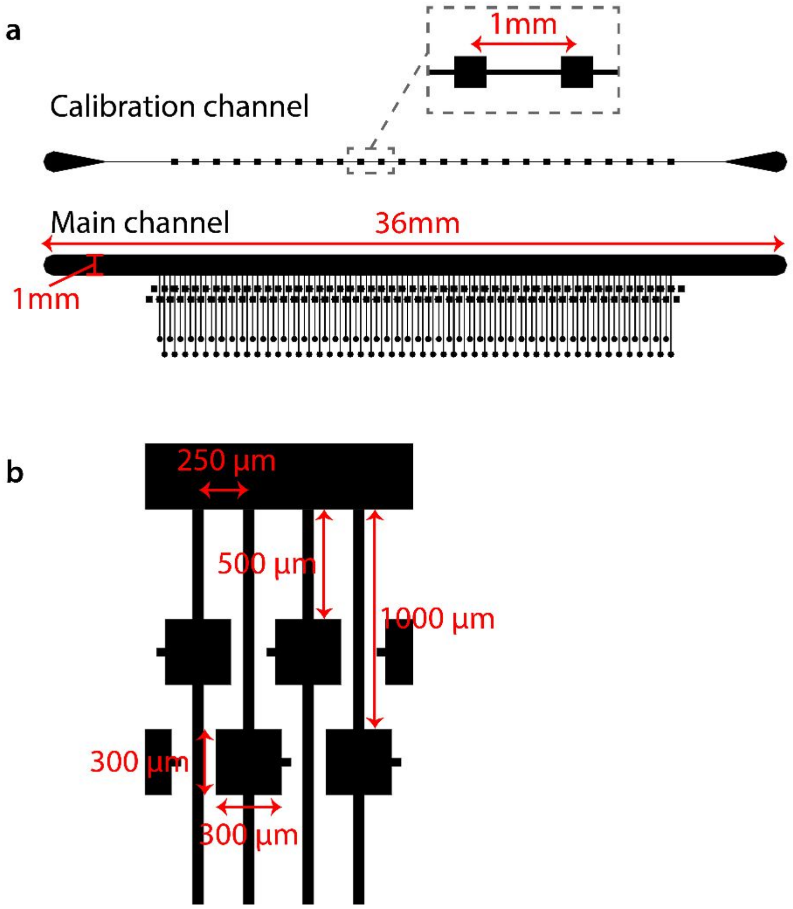


**Fig. S2:** Schematic design of a) the microfluidic device and of b) the analysis chambers used in the single molecule microarray experiments. The height of the microchannels was 32 µm. The total volume of each chamber was approximately 2.9 nL.

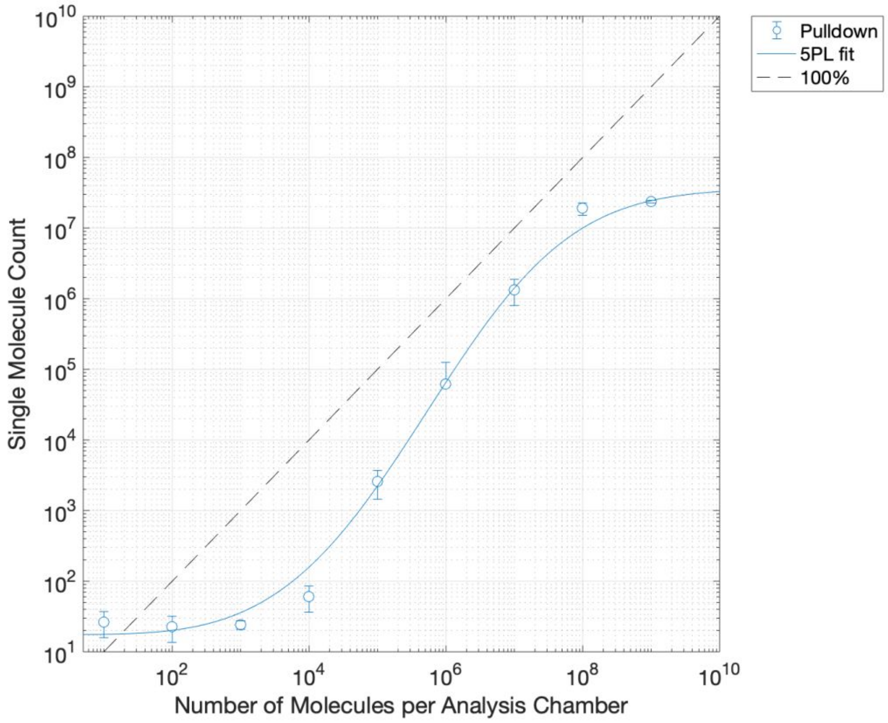


**Fig. S3:** A calibration curve was obtained using standard solutions of streptavidin Alexa Fluor 488 of known concentration. Solutions were flowed into the calibration channel of the microfluidic device and the number of single molecules were counted per spot. It is used to calibrate the single molecule counted per spot to the number of proteins per GUV.

| Method of Segmentation | GUV volume in pL (average ± standard deviation) |
|---|---|
| Manual (brightfield channel) / Ground truth | 2.00 ± 3.32 |
| Manual (fluorescence channel) | 2.09 ± 3.72 |
| Automatic (CellProfiler) | 13.8 ± 24.8 |

**Table S1:** Large field images were processed manually in FiJi and automatically using CellProfiler v2.2.0. The size of each segmented GUV was used to calculate the spherical volume of the GUV. The volume calculated using images of GUVs in brightfield were considered the ground truth against which manual and automatic segmentation in the fluorescence channel were compared. The difference / error in average volume calculated from images manually segmented in the fluorescence channel compared to the brightfield was 4.5%.
